# Supplementary material for: No Impact of Seasonality of Diagnoses on Baseline Tumor Immune Infiltration, Response to Treatment, and Prognosis in BC Patients Treated with NAC
Source: Cancers (Basel). 2022 Jun 23;14(13):3080. doi: 10.3390/cancers14133080 (PMC9264787; doi:10.3390/cancers14133080)
Supplement: Supplementary file 1 [file cancers-14-03080-s001.zip › cancers-1746642-supplementary.pdf]

SUPPLEMENTARY FIGURES

Supplementary Figure S1. Associations between pre-NAC TILs and month in whole population, and after stratification by breast cancer subtype. Bottomand top bars of the boxplots represent the first and third quartiles, respectively, the medium bar is the median, and whiskers extend to 1.5 times the interquartile range. **A,** Pre-NAC str TIL levels according to month. **B,** Pre-NAC IT TIL levels according to month. **C,** Pre-NAC str TIL levels according to month and breast cancer subtype. **D,** Pre-NAC IT TIL levels according to month and breast cancer subtype.

Supplementary Figure S2. Barplot of associations between response to treatment and season at breast cancer diagnosis in the whole population, and by chemotherapy regimen. **A,** among the whole population. **B,** by chemotherapy regimen.

Supplementary Figure S3. RCB class distribution among the whole population and by season at breast cancer diagnosis. **A,** among the whole population. **B,** in each season.

Supplementary Figure S4. Post-NAC TIL levels by season at BC diagnosis in the general population and by breast cancer subtype. Differences among seasons. The first and third quartiles are represented by the bottom and top bars of the boxplots, respectively; the median is represented by the medium bar, and whiskers extend to 1.5 times the interquartile range. **A,** stromal lymphocytes among the whole population. **B,** stromal lymphocytes in each BC subtype.

Supplementary Figure S5. Associations between post-NAC TILs and month in whole population, and after stratification by breast cancer subtype. Bottomand top bars of the boxplots represent the first and third quartiles, respectively, the medium bar is the median, and whiskers extend to 1.5 times the interquartile range. **A,** Post-NAC str TIL levels according to month. **B,** Post-NAC IT TIL levels according to month. **C,** Post-NAC str TIL levels according to month and breast cancer subtype. **D,** Post-NAC IT TIL levels according to month and breast cancer subtype.

Supplementary Figure S6. Distant relapse-free survival curves by season at BC diagnosis. **A,** Distant relapse-free survival curves according to season. **B,** Distant relapse-free survival curves in Luminal breast cancer according to season. **C,** Distant relapse-free survival curves in TNBC breast cancer according to season. **D,** Distant relapse-free survival curves in Her2-positive breast cancer according to season.

Supplementary Figure S7. Forest plot – Hazard Ratio RFS (Univariate analysis). Survival impact of neoadjuvant chemotherapy (NAC) strategy according to variables – Recurrence Free Survival (RFS).

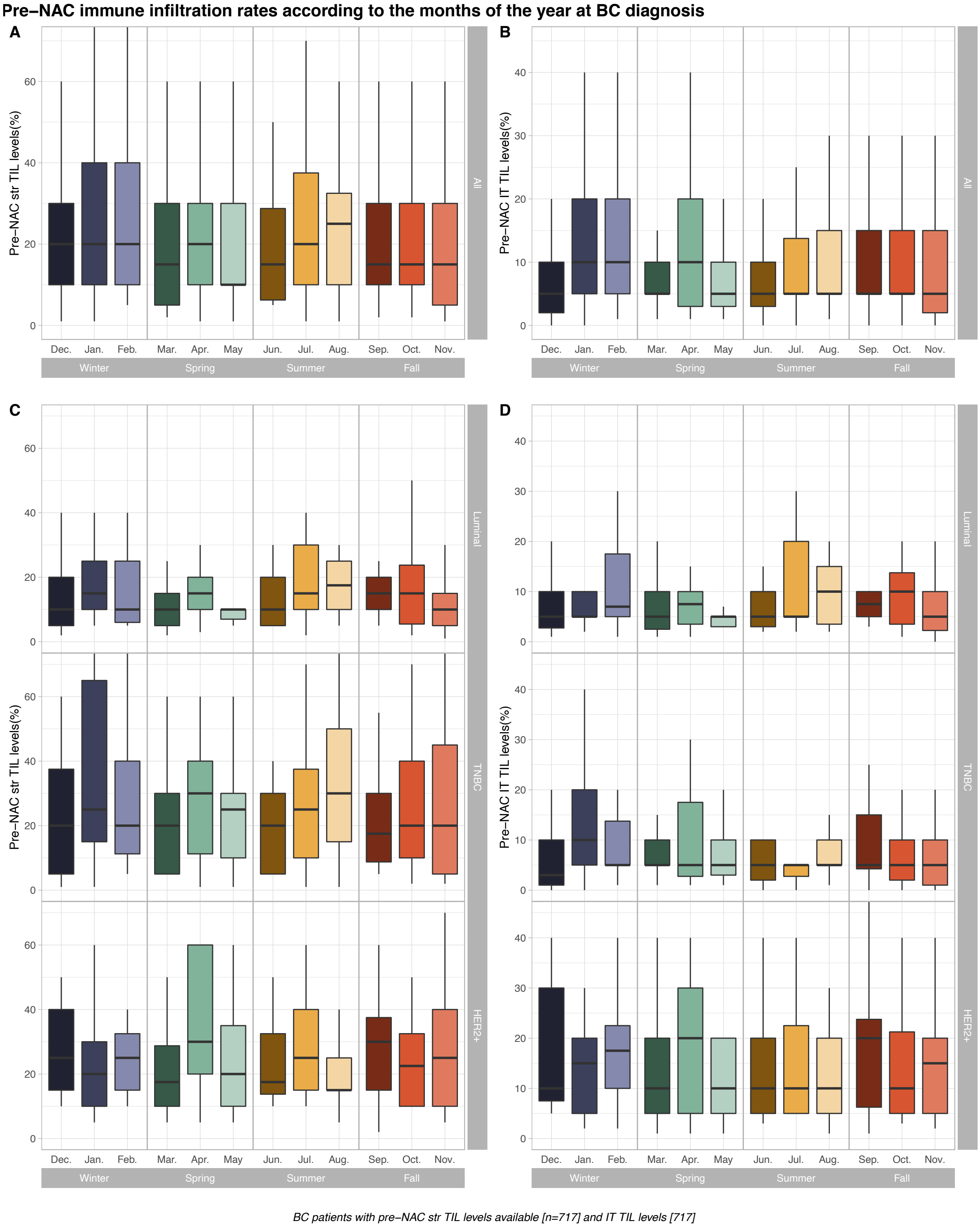

Supplementary Figure S1. *Associations between pre-NAC TILs and month in whole population, and after stratification by breast cancer subtype. Bottomand top bars of the boxplots represent the first and third quartiles, respectively, the medium bar is the median, and whiskers extend to 1.5 times the interquartile range.* ***A****, Pre-NAC str TIL levels according to month.* ***B,*** *Pre-NAC IT TIL levels according to month.* ***C,*** *Pre-NAC str TIL levels according to month and breast cancer subtype.* ***D****, Pre-NAC IT TIL levels according to month and breast cancer subtype.*

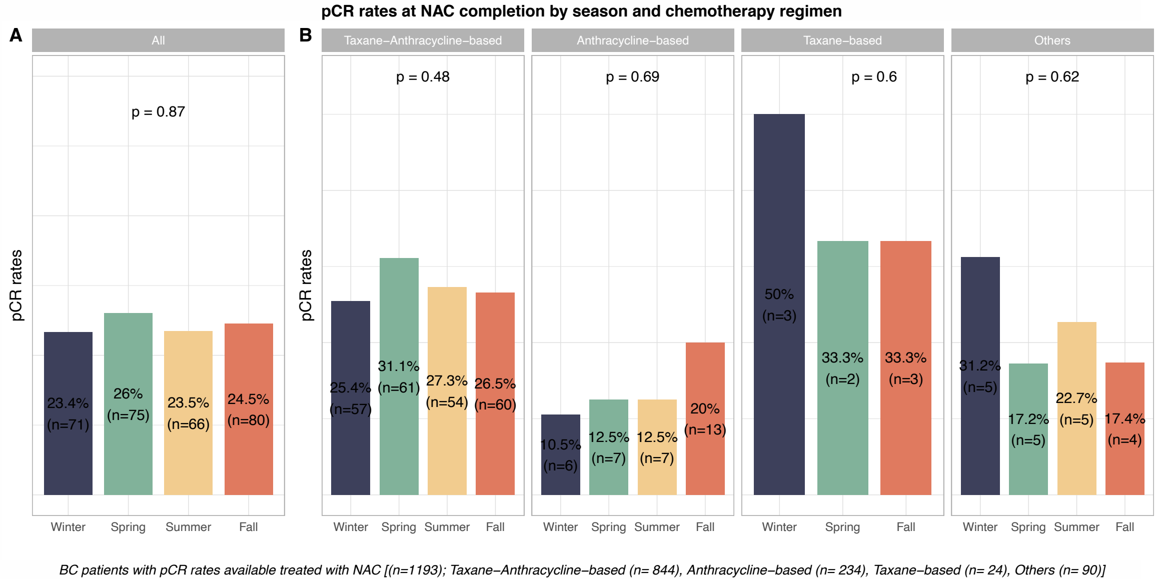

Supplementary Figure S2. *Barplot of associations between response to treatment and season at breast cancer diagnosis in the whole population, and by chemotherapy regimen.* ***A****, among the whole population.* ***B****, by chemotherapy regimen.*

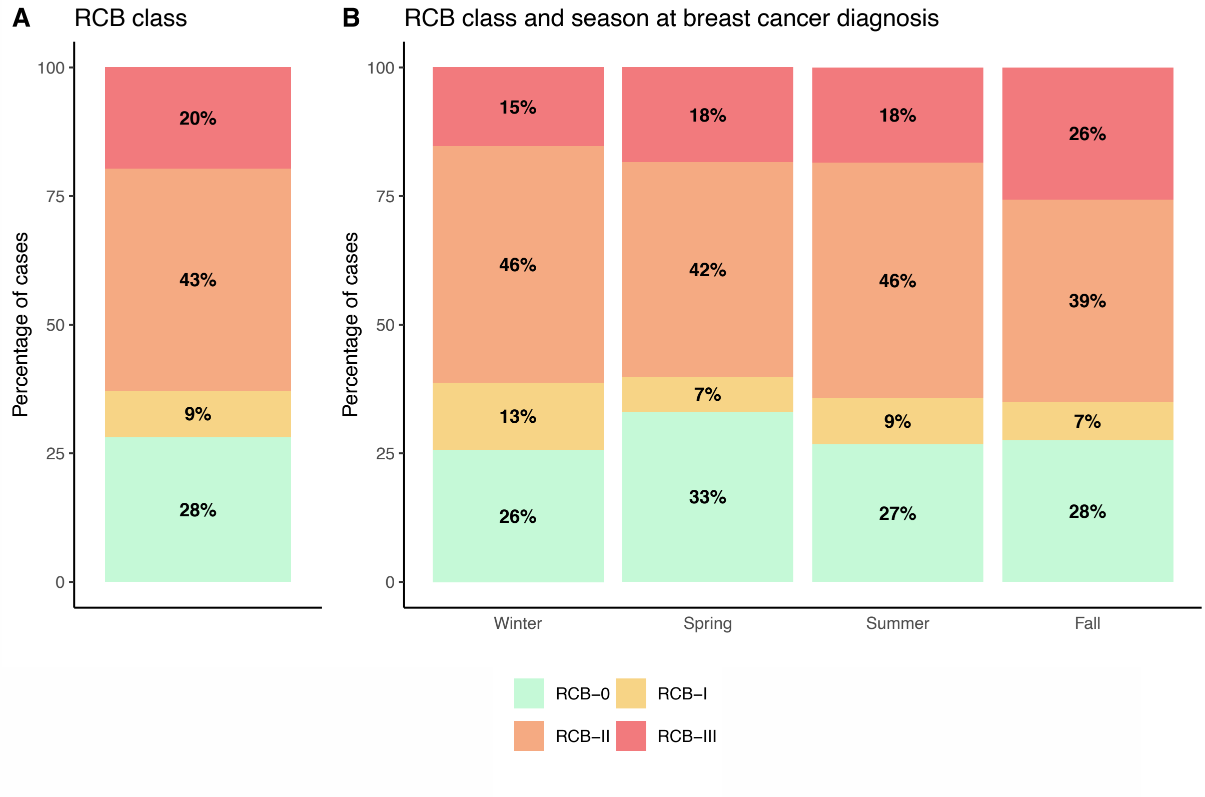

Supplementary Figure S3*. RCB class distribution among the whole population and by season at breast cancer diagnosis.* ***A****, among the whole population.* ***B,*** *in each season.*

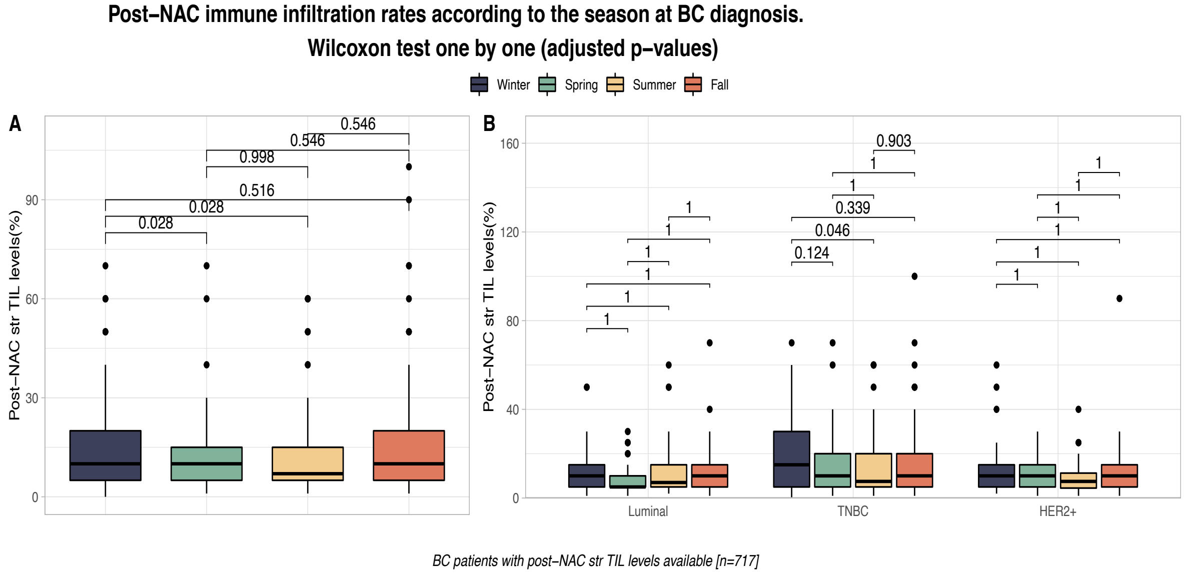

Supplementary Figure S4. *Post-NAC TIL levels by season at BC diagnosis in the general population and by breast cancer subtype. Differences among seasons. The first and third quartiles are represented by the bottom and top bars of the boxplots, respectively; the median is represented by the medium bar, and whiskers extend to 1.5 times the interquartile range.* ***A,*** *stromal lymphocytes among the whole population.* ***B,*** *stromal lymphocytes in each BC subtype.*

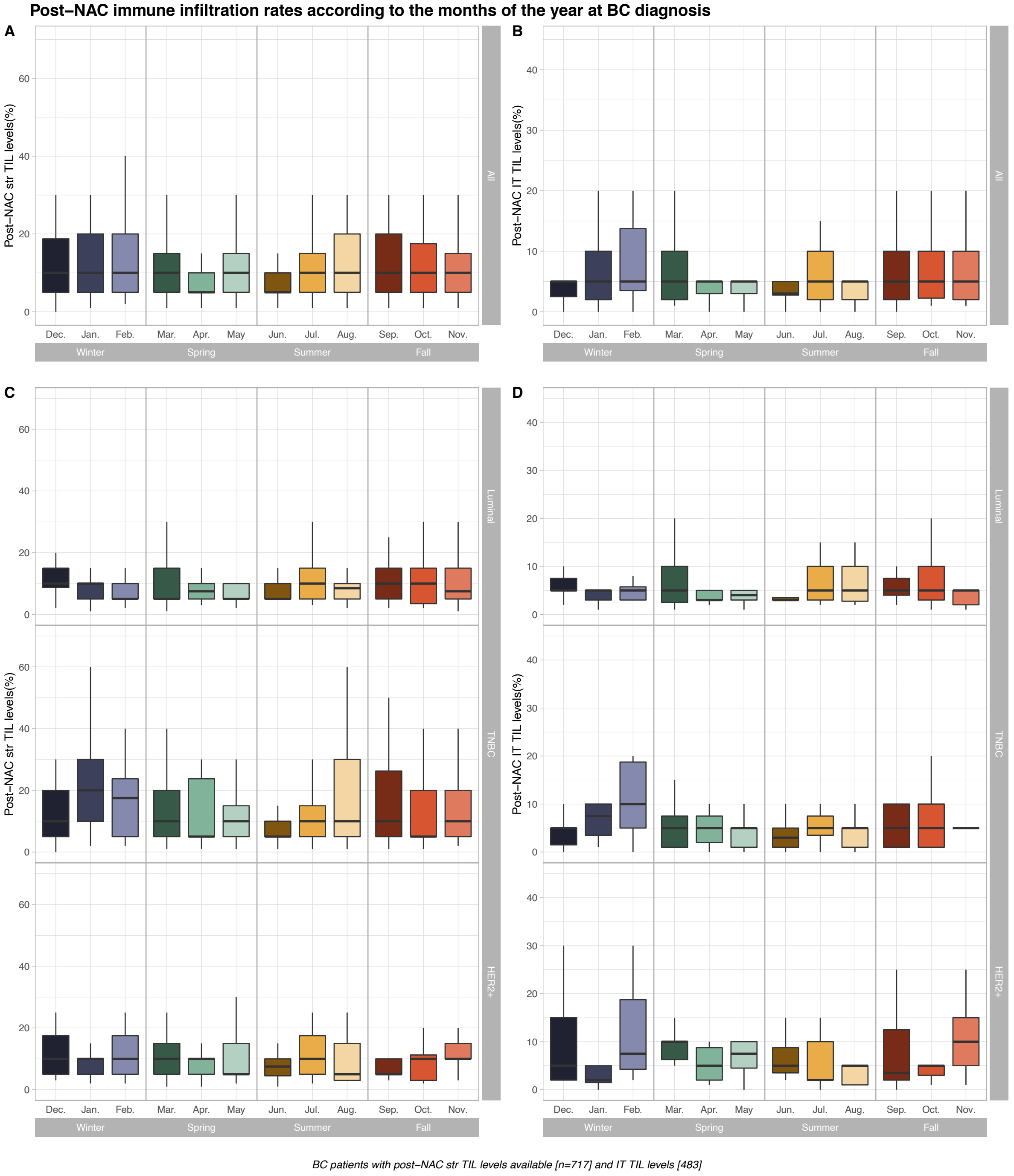

Supplementary Figure S5. *Associations between post-NAC TILs and month in whole population, and after stratification by breast cancer subtype. Bottomand top bars of the boxplots represent the first and third quartiles, respectively, the medium bar is the median, and whiskers extend to 1.5 times the interquartile range.* ***A,*** *Post-NAC str TIL levels according to month.* ***B,*** *Post-NAC IT TIL levels according to month.* ***C,*** *Post-NAC str TIL levels according to month and breast cancer subtype.* ***D,*** *Post-NAC IT TIL levels according to month and breast cancer subtype.*

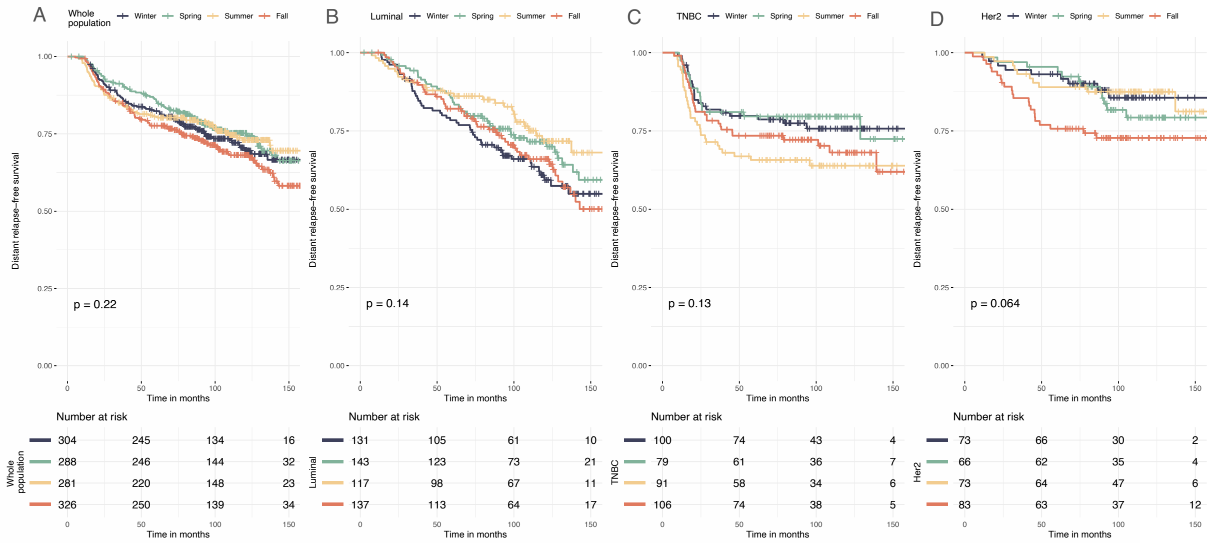

Supplementary Figure S6. *Distant relapse-free survival curves by season at BC diagnosis.* ***A,*** *Distant relapse-free survival curves according to season.* ***B,*** *Distant relapse-free survival curves in Luminal breast cancer according to season.* ***C,*** *Distant relapse-free survival curves in TNBC breast cancer according to season.* ***D,*** *Distant relapse-free survival curves in Her2-positive breast cancer according to season.*

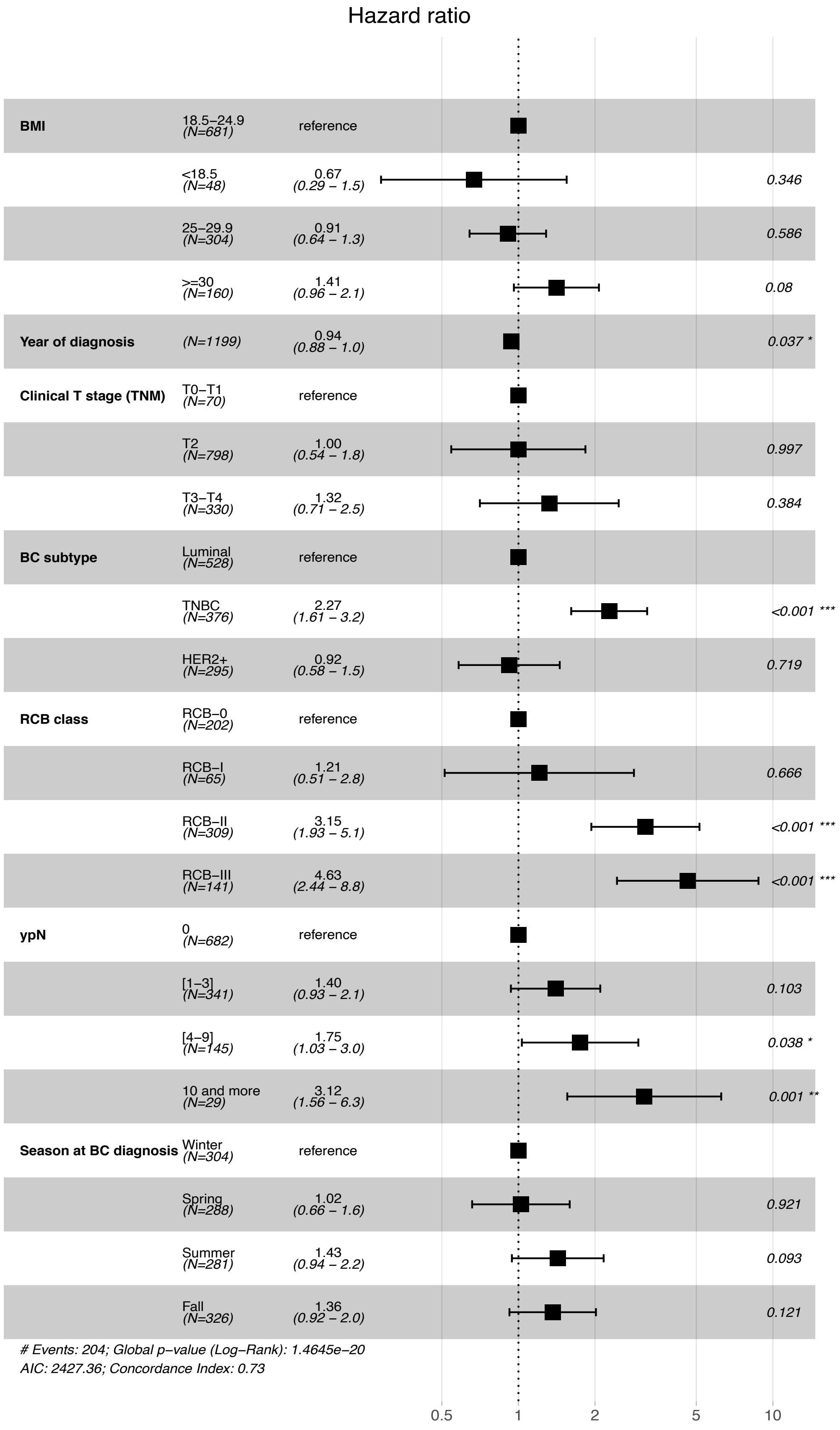

Supplementary Figure S7. *Forest plot – Hazard Ratio RFS (Univariate analysis). Survival impact of neoadjuvant chemotherapy (NAC) strategy according to variables – Recurrence Free Survival (RFS).*

SUPPLEMENTARY TABLES

Supplementary Table S1. Patients ‘characteristics according to season in each tumor subtype.

Missing data: Missing data: Menopausal status, n=10; BMI, n=6; Smoking status, n=243; Hereditary predisposition, n=932; Clinical Tumor size (mm), n=1; Clinical T stage (TNM), n=1; Clinical N stage (TNM), n=1; SBR grade, n=42; KI67, n=617; Mitotic index, n=118; DCIS component, n=206; Stromal TIL levels (%), n=482; IT TIL levels (%), n=482; LVI, n=761; Histological type, n=63; Pathological complete response, n=6; RCB index (continuous), n=482; Residual Cancer Burden class, n=482; ypN, n=2; Stromal TIL levels (%) (post-NAC), n=482; IT TIL levels (%) (post-NAC), n=716.

Abbreviations: BMI=body mass index; NST= no special type; TNBC= triple negative breast cancer; str TILs= stromal tumor-infiltrating lymphocytes; IT TILs= intratumoral-infiltrating lymphocytes; pCR=Pathologic complete response. *The "n" denotes the number of patients. In case of categorical variables, percentages are expressed between brackets. In case of continuous variables, mean value is reported. In case of nonnormal continuous variables, median value is reported, with interquartile range between brackets.*

Supplementary Table S2. Association of season with pCR after univariate analysis in the whole population.

Missing data: Missing data: Menopausal status, n=10; BMI, n=6; Smoking status, n=243; Hereditary predisposition, n=932; Clinical Tumor size (mm), n=1; Clinical T stage (TNM), n=1; Clinical N stage (TNM), n=1; SBR grade, n=42; KI67, n=617; Mitotic index, n=118; DCIS component, n=206; Stromal TIL levels (%), n=482; IT TIL levels (%), n=482; LVI, n=761; Histological type, n=63; Pathological complete response, n=6; RCB index (continuous), n=482; Residual Cancer Burden class, n=482; ypN, n=2; Stromal TIL levels (%) (post-NAC), n=482; IT TIL levels (%) (post-NAC), n=716.

Abbreviations: BMI=body mass index; NST= no special type; TNBC= triple negative breast cancer; str TILs= stromal tumor-infiltrating lymphocytes; IT TILs= intratumoral-infiltrating lymphocytes; pCR=Pathologic complete response. *The "n" denotes the number of patients. In case of categorical variables, percentages are expressed between brackets. In case of continuous variables, mean value is reported. In case of nonnormal continuous variables, median value is reported, with interquartile range between brackets.*

Supplementary Table S3. Literature Review

Abbreviations: PR= progesterone receptor; ER= estrogen receptor; HR= hormone receptor; BC= breast cancer; HR= hazard ratio.

Luminal

	Class	Winter	Spring	Summer	Fall	p	test
	n	131	143	117	137		
Age at BC diagnosis		48.40 [40.45, 54.10]	47.40 [42.35, 53.05]	49.70 [44.00, 56.70]	48.20 [42.50, 56.60]	0.182	nonnorm
Menopausal status	Premenopausal	89 (68.5)	98 (69.5)	74 (63.2)	78 (57.8)	0.159	
	Postmenopausal	41 (31.5)	43 (30.5)	43 (36.8)	57 (42.2)		
BMI	18.5-24.9	72 (55.0)	80 (56.3)	65 (56.0)	73 (53.7)	0.535	
	<18.5	2 (1.5)	7 (4.9)	7 (6.0)	8 (5.9)		
	25-29.9	34 (26.0)	36 (25.4)	33 (28.4)	32 (23.5)		
	>=30	23 (17.6)	19 (13.4)	11 (9.5)	23 (16.9)		
BMI		24.30 [21.65, 28.20]	23.60 [21.52, 26.87]	23.50 [21.40, 26.63]	23.70 [20.90, 28.00]	0.483	nonnorm
Smoking status	Never	73 (68.2)	68 (61.3)	55 (63.2)	59 (56.2)	0.053	
	Current	21 (19.6)	22 (19.8)	12 (13.8)	32 (30.5)		
	Former	13 (12.1)	21 (18.9)	20 (23.0)	14 (13.3)		
Year BC diagnosis		2006.83 (2.66)	2006.21 (2.60)	2006.12 (2.41)	2006.16 (2.38)	0.074	
Hereditary predisposition	No	24 (88.9)	19 (86.4)	13 (72.2)	19 (82.6)	0.502	
	Yes	3 (11.1)	3 (13.6)	5 (27.8)	4 (17.4)		
Clinical Tumor size (mm)		45.00 [35.00, 60.00]	40.00 [30.00, 50.00]	42.50 [40.00, 55.00]	45.00 [35.00, 55.00]	0.531	nonnorm
Clinical T stage (TNM)	T0-T1	6 (4.6)	7 (4.9)	2 (1.7)	6 (4.4)	0.525	
	T2	82 (62.6)	101 (70.6)	85 (72.6)	91 (66.4)		
	T3-T4	43 (32.8)	35 (24.5)	30 (25.6)	40 (29.2)		
Clinical N stage (TNM)	N0	52 (39.7)	71 (49.7)	59 (50.9)	53 (38.7)	0.088	
	N1-N2-N3	79 (60.3)	72 (50.3)	57 (49.1)	84 (61.3)		
SBR grade	Grade I	9 (7.2)	16 (11.5)	10 (8.9)	5 (3.8)	0.158	
	Grade II	68 (54.4)	80 (57.6)	67 (59.8)	71 (53.8)		
	Grade III	48 (38.4)	43 (30.9)	35 (31.2)	56 (42.4)		
KI67		20.00 [12.00, 40.00]	20.50 [11.25, 40.00]	20.00 [14.00, 35.00]	20.00 [11.00, 32.00]	0.854	nonnorm
KI67	[0-10)	9 (12.7)	17 (20.7)	13 (20.6)	13 (17.8)	0.850	
	[10-20)	21 (29.6)	21 (25.6)	15 (23.8)	22 (30.1)		
	>=20	41 (57.7)	44 (53.7)	35 (55.6)	38 (52.1)		
Mitotic index		11.00 [4.00, 21.75]	8.00 [4.00, 16.50]	10.00 [4.00, 15.25]	11.00 [4.00, 22.00]	0.507	nonnorm
Mitotic index	[0-7) mitose/2 mm2	50 (45.5)	69 (52.7)	51 (49.0)	59 (47.2)	0.510	
	[7-13) mitose/2 mm2	31 (28.2)	31 (23.7)	34 (32.7)	31 (24.8)		
	>=13 mitose ou plus/2 mm2.	29 (26.4)	31 (23.7)	19 (18.3)	35 (28.0)		
DCIS component	No	44 (44.4)	42 (37.8)	41 (46.6)	59 (52.7)	0.169	
	Yes	55 (55.6)	69 (62.2)	47 (53.4)	53 (47.3)		
Stromal TIL levels (%)		10.00 [6.50, 21.25]	10.00 [5.00, 15.00]	10.00 [10.00, 25.00]	15.00 [5.00, 20.00]	0.411	nonnorm
Stromal TIL levels (%)	[0 -30[	40 (76.9)	51 (94.4)	39 (81.2)	58 (85.3)	0.077	
	>=30	12 (23.1)	3 (5.6)	9 (18.8)	10 (14.7)		
IT TIL levels (%)		5.00 [5.00, 11.25]	5.00 [3.00, 10.00]	5.00 [3.00, 15.00]	5.00 [3.00, 10.00]	0.621	nonnorm
IT TIL levels (%)	[0,10]	39 (75.0)	46 (85.2)	33 (68.8)	53 (77.9)	0.257	
	(10,100]	13 (25.0)	8 (14.8)	15 (31.2)	15 (22.1)		
LVI	No	34 (64.2)	42 (66.7)	34 (59.6)	34 (61.8)	0.873	
	Yes	19 (35.8)	21 (33.3)	23 (40.4)	21 (38.2)		
Histological type	NST	114 (89.1)	116 (86.6)	101 (87.1)	115 (86.5)	0.916	
	Others	14 (10.9)	18 (13.4)	15 (12.9)	18 (13.5)		
Pathological complete response	No	121 (93.1)	132 (93.0)	109 (93.2)	130 (94.9)	0.904	
	Yes	9 (6.9)	10 (7.0)	8 (6.8)	7 (5.1)		
RCB index (continuous)		2.53 [1.61, 3.46]	2.99 [1.85, 3.52]	3.01 [1.68, 3.39]	3.23 [2.09, 3.56]	0.379	nonnorm
Residual Cancer Burden class	RCB-0	2 (3.8)	3 (5.6)	3 (6.2)	3 (4.4)	0.660	
	RCB-I	7 (13.5)	3 (5.6)	2 (4.2)	6 (8.8)		
	RCB-II	25 (48.1)	30 (55.6)	26 (54.2)	28 (41.2)		
	RCB-III	18 (34.6)	18 (33.3)	17 (35.4)	31 (45.6)		
ypN	0	51 (39.2)	53 (37.3)	51 (43.6)	42 (30.7)	0.664	
	[1-3]	47 (36.2)	59 (41.5)	46 (39.3)	64 (46.7)		
	[4-9]	26 (20.0)	25 (17.6)	16 (13.7)	25 (18.2)		
	10 and more	6 (4.6)	5 (3.5)	4 (3.4)	6 (4.4)		
Stromal TIL levels (%) (post-NAC)		10.00 [5.00, 15.00]	5.00 [5.00, 10.00]	7.00 [5.00, 15.00]	10.00 [5.00, 15.00]	0.622	nonnorm
Stromal TIL levels (%) (post-NAC)	[0,10]	38 (73.1)	44 (81.5)	35 (72.9)	44 (64.7)	0.234	
	(10,100]	14 (26.9)	10 (18.5)	13 (27.1)	24 (35.3)		
IT TIL levels (%) (post-NAC)		5.00 [3.00, 5.00]	3.00 [3.00, 5.00]	3.00 [3.00, 10.00]	5.00 [2.50, 10.00]	0.411	nonnorm
IT TIL levels (%) (post-NAC)	[0,10]	38 (84.4)	41 (89.1)	39 (86.7)	54 (85.7)	0.926	
	(10,100]	7 (15.6)	5 (10.9)	6 (13.3)	9 (14.3)		

TNBC

	Class	Winter	Spring	Summer	Fall	p	test
	n	100	79	91	106		
Age at BC diagnosis		46.65 [40.77, 54.02]	48.50 [39.15, 58.30]	50.10 [38.90, 57.50]	47.35 [41.92, 54.43]	0.864	nonnorm
Menopausal status	Premenopausal	64 (64.6)	46 (59.0)	50 (54.9)	69 (66.3)	0.346	
	Postmenopausal	35 (35.4)	32 (41.0)	41 (45.1)	35 (33.7)		
BMI	18.5-24.9	52 (52.5)	47 (59.5)	52 (57.1)	60 (56.6)	0.518	
	<18.5	5 (5.1)	0 (0.0)	4 (4.4)	3 (2.8)		
	25-29.9	33 (33.3)	21 (26.6)	23 (25.3)	26 (24.5)		
	>=30	9 (9.1)	11 (13.9)	12 (13.2)	17 (16.0)		
BMI		23.70 [21.10, 26.75]	23.90 [21.70, 26.90]	24.20 [21.70, 27.65]	23.85 [21.52, 27.85]	0.828	nonnorm
Smoking status	Never	57 (70.4)	39 (60.0)	51 (71.8)	59 (67.0)	0.334	
	Current	16 (19.8)	14 (21.5)	10 (14.1)	11 (12.5)		
	Former	8 (9.9)	12 (18.5)	10 (14.1)	18 (20.5)		
Year BC diagnosis		2007.39 (2.79)	2007.80 (2.46)	2006.54 (2.60)	2007.16 (2.55)	**0.016**	
Hereditary predisposition	No	20 (62.5)	19 (79.2)	21 (80.8)	23 (82.1)	0.246	
	Yes	12 (37.5)	5 (20.8)	5 (19.2)	5 (17.9)		
Clinical Tumor size (mm)		40.00 [30.00, 50.00]	40.00 [30.00, 50.00]	40.00 [32.50, 60.00]	45.00 [35.00, 60.00]	0.112	nonnorm
Clinical T stage (TNM)	T0-T1	8 (8.0)	4 (5.1)	12 (13.2)	7 (6.6)	0.056	
	T2	72 (72.0)	57 (72.2)	48 (52.7)	66 (62.3)		
	T3-T4	20 (20.0)	18 (22.8)	31 (34.1)	33 (31.1)		
Clinical N stage (TNM)	N0	40 (40.0)	42 (53.2)	40 (44.0)	49 (46.2)	0.362	
	N1-N2-N3	60 (60.0)	37 (46.8)	51 (56.0)	57 (53.8)		
SBR grade	Grade I	2 (2.0)	1 (1.3)	2 (2.3)	0 (0.0)	0.787	
	Grade II	14 (14.3)	8 (10.3)	11 (12.6)	15 (14.4)		
	Grade III	82 (83.7)	69 (88.5)	74 (85.1)	89 (85.6)		
KI67		50.00 [28.50, 76.25]	60.00 [30.00, 72.50]	47.50 [15.75, 70.00]	70.00 [40.00, 80.00]	0.077	nonnorm
KI67	[0-10)	1 (2.3)	3 (8.6)	6 (13.6)	0 (0.0)	0.066	
	[10-20)	5 (11.4)	3 (8.6)	6 (13.6)	2 (4.5)		
	>=20	38 (86.4)	29 (82.9)	32 (72.7)	42 (95.5)		
Mitotic index		27.00 [13.00, 41.00]	27.00 [17.00, 45.00]	24.00 [15.00, 40.00]	27.50 [14.00, 40.00]	0.830	nonnorm
Mitotic index	[0-7) mitose/2 mm2	8 (9.0)	10 (13.2)	10 (13.0)	12 (11.8)	0.679	
	[7-13) mitose/2 mm2	23 (25.8)	11 (14.5)	17 (22.1)	24 (23.5)		
	>=13 mitose ou plus/2 mm2.	58 (65.2)	55 (72.4)	50 (64.9)	66 (64.7)		
DCIS component	No	80 (88.9)	59 (83.1)	69 (85.2)	74 (81.3)	0.537	
	Yes	10 (11.1)	12 (16.9)	12 (14.8)	17 (18.7)		
Stromal TIL levels (%)		20.00 [10.00, 40.00]	25.00 [10.00, 35.00]	25.00 [10.00, 40.00]	20.00 [9.25, 40.00]	0.791	nonnorm
Stromal TIL levels (%)	[0 -30[	50 (59.5)	36 (53.7)	44 (55.0)	53 (60.2)	0.801	
	>=30	34 (40.5)	31 (46.3)	36 (45.0)	35 (39.8)		
IT TIL levels (%)		5.00 [2.00, 15.00]	5.00 [4.00, 10.00]	5.00 [2.75, 6.25]	5.00 [2.00, 10.00]	0.643	nonnorm
IT TIL levels (%)	[0,10]	61 (72.6)	53 (79.1)	70 (87.5)	68 (77.3)	0.127	
	(10,100]	23 (27.4)	14 (20.9)	10 (12.5)	20 (22.7)		
LVI	No	18 (62.1)	15 (62.5)	24 (70.6)	25 (75.8)	0.610	
	Yes	11 (37.9)	9 (37.5)	10 (29.4)	8 (24.2)		
Histological type	NST	94 (98.9)	75 (98.7)	79 (97.5)	92 (97.9)	0.878	
	Others	1 (1.1)	1 (1.3)	2 (2.5)	2 (2.1)		
Pathological complete response	No	68 (68.0)	42 (53.2)	56 (62.9)	65 (61.3)	0.243	
	Yes	32 (32.0)	37 (46.8)	33 (37.1)	41 (38.7)		
RCB index (continuous)		1.67 [0.00, 2.54]	1.33 [0.00, 2.23]	1.44 [0.00, 2.34]	1.63 [0.00, 2.32]	0.667	nonnorm
Residual Cancer Burden class	RCB-0	28 (33.3)	30 (44.8)	29 (36.2)	36 (40.9)	0.329	
	RCB-I	6 (7.1)	4 (6.0)	10 (12.5)	3 (3.4)		
	RCB-II	42 (50.0)	24 (35.8)	29 (36.2)	36 (40.9)		
	RCB-III	8 (9.5)	9 (13.4)	12 (15.0)	13 (14.8)		
ypN	0	70 (70.0)	61 (77.2)	71 (78.0)	78 (73.6)	0.759	
	[1-3]	19 (19.0)	9 (11.4)	9 (9.9)	18 (17.0)		
	[4-9]	10 (10.0)	7 (8.9)	8 (8.8)	8 (7.5)		
	10 and more	1 (1.0)	2 (2.5)	3 (3.3)	2 (1.9)		
Stromal TIL levels (%) (post-NAC)		15.00 [5.00, 30.00]	10.00 [5.00, 20.00]	7.50 [5.00, 20.00]	10.00 [5.00, 20.00]	**0.035**	nonnorm
Stromal TIL levels (%) (post-NAC)	[0,10]	36 (42.4)	42 (62.7)	55 (68.8)	55 (63.2)	**0.003**	
	(10,100]	49 (57.6)	25 (37.3)	25 (31.2)	32 (36.8)		
IT TIL levels (%) (post-NAC)		5.00 [2.00, 10.00]	5.00 [1.00, 5.00]	5.00 [1.00, 5.00]	5.00 [1.00, 10.00]	0.199	nonnorm
IT TIL levels (%) (post-NAC)	[0,10]	44 (86.3)	33 (94.3)	46 (95.8)	41 (91.1)	0.341	
	(10,100]	7 (13.7)	2 (5.7)	2 (4.2)	4 (8.9)		

HER2-positive

	Class	Winter	Spring	Summer	Fall	p	test
	n	73	66	73	83		
Age at BC diagnosis		45.90 [37.90, 53.80]	48.25 [40.38, 58.60]	48.30 [40.50, 57.40]	47.90 [39.70, 56.10]	0.661	nonnorm
Menopausal status	Premenopausal	47 (64.4)	38 (58.5)	42 (57.5)	52 (62.7)	0.804	
	Postmenopausal	26 (35.6)	27 (41.5)	31 (42.5)	31 (37.3)		
BMI	18.5-24.9	49 (68.1)	44 (66.7)	39 (54.2)	48 (57.8)	0.254	
	<18.5	2 (2.8)	3 (4.5)	1 (1.4)	6 (7.2)		
	25-29.9	15 (20.8)	15 (22.7)	19 (26.4)	17 (20.5)		
	>=30	6 (8.3)	4 (6.1)	13 (18.1)	12 (14.5)		
BMI		23.30 [21.15, 25.22]	22.80 [21.25, 26.15]	24.35 [22.48, 28.25]	23.40 [21.05, 27.50]	0.121	nonnorm
Smoking status	Never	45 (69.2)	35 (70.0)	35 (58.3)	47 (71.2)	0.593	
	Current	11 (16.9)	7 (14.0)	15 (25.0)	8 (12.1)		
	Former	9 (13.8)	8 (16.0)	10 (16.7)	11 (16.7)		
Year BC diagnosis		2008.18 (2.28)	2007.59 (2.54)	2006.68 (2.24)	2006.73 (2.60)	**<0.001**	
Hereditary predisposition	No	14 (93.3)	17 (94.4)	14 (93.3)	18 (94.7)	0.997	
	Yes	1 (6.7)	1 (5.6)	1 (6.7)	1 (5.3)		
Clinical Tumor size (mm)		40.00 [30.00, 55.00]	40.00 [30.00, 50.00]	40.00 [30.00, 50.00]	40.00 [30.00, 60.00]	0.835	nonnorm
Clinical T stage (TNM)	T0-T1	5 (6.8)	2 (3.1)	6 (8.2)	5 (6.0)	0.681	
	T2	47 (64.4)	49 (75.4)	48 (65.8)	52 (62.7)		
	T3-T4	21 (28.8)	14 (21.5)	19 (26.0)	26 (31.3)		
Clinical N stage (TNM)	N0	28 (38.4)	28 (42.4)	31 (42.5)	32 (38.6)	0.922	
	N1-N2-N3	45 (61.6)	38 (57.6)	42 (57.5)	51 (61.4)		
SBR grade	Grade I	0 (0.0)	0 (0.0)	1 (1.5)	1 (1.3)	0.390	
	Grade II	25 (34.2)	16 (25.0)	26 (38.2)	31 (40.3)		
	Grade III	48 (65.8)	48 (75.0)	41 (60.3)	45 (58.4)		
KI67		26.00 [21.00, 35.00]	35.00 [26.50, 44.25]	40.00 [30.50, 62.50]	40.00 [25.00, 60.00]	**0.018**	nonnorm
KI67	[0-10)	2 (7.4)	0 (0.0)	1 (3.3)	0 (0.0)	0.518	
	[10-20)	4 (14.8)	3 (10.7)	3 (10.0)	5 (12.2)		
	>=20	21 (77.8)	25 (89.3)	26 (86.7)	36 (87.8)		
Mitotic index		14.00 [8.00, 23.50]	18.00 [12.00, 25.00]	16.00 [11.00, 25.00]	15.00 [6.75, 24.00]	0.150	nonnorm
Mitotic index	[0-7) mitose/2 mm2	22 (31.0)	10 (16.4)	15 (23.8)	25 (34.7)	0.222	
	[7-13) mitose/2 mm2	27 (38.0)	22 (36.1)	23 (36.5)	21 (29.2)		
	>=13 mitose ou plus/2 mm2.	22 (31.0)	29 (47.5)	25 (39.7)	26 (36.1)		
DCIS component	No	35 (51.5)	29 (49.2)	32 (50.0)	40 (67.8)	0.129	
	Yes	33 (48.5)	30 (50.8)	32 (50.0)	19 (32.2)		
Stromal TIL levels (%)		20.00 [15.00, 40.00]	25.00 [10.00, 40.00]	20.00 [15.00, 32.50]	25.00 [10.00, 40.00]	0.973	nonnorm
Stromal TIL levels (%)	[0 -30[	26 (55.3)	25 (59.5)	27 (67.5)	26 (55.3)	0.631	
	>=30	21 (44.7)	17 (40.5)	13 (32.5)	21 (44.7)		
IT TIL levels (%)		15.00 [5.00, 27.50]	12.50 [5.00, 20.00]	10.00 [5.00, 20.00]	15.00 [5.00, 20.00]	0.796	nonnorm
IT TIL levels (%)	[0,10]	22 (46.8)	21 (50.0)	23 (57.5)	22 (46.8)	0.733	
	(10,100]	25 (53.2)	21 (50.0)	17 (42.5)	25 (53.2)		
LVI	No	9 (42.9)	12 (54.5)	11 (47.8)	9 (37.5)	0.693	
	Yes	12 (57.1)	10 (45.5)	12 (52.2)	15 (62.5)		
Histological type	NST	68 (98.6)	65 (100.0)	67 (97.1)	76 (100.0)	0.286	
	Others	1 (1.4)	0 (0.0)	2 (2.9)	0 (0.0)		
Pathological complete response	No	43 (58.9)	38 (57.6)	48 (65.8)	49 (60.5)	0.765	
	Yes	30 (41.1)	28 (42.4)	25 (34.2)	32 (39.5)		
RCB index (continuous)		1.14 [0.00, 1.94]	0.42 [0.00, 1.72]	1.61 [0.00, 2.32]	1.44 [0.00, 2.81]	0.143	nonnorm
Residual Cancer Burden class	RCB-0	17 (36.2)	21 (50.0)	13 (32.5)	17 (36.2)	0.069	
	RCB-I	11 (23.4)	4 (9.5)	3 (7.5)	6 (12.8)		
	RCB-II	17 (36.2)	14 (33.3)	22 (55.0)	16 (34.0)		
	RCB-III	2 (4.3)	3 (7.1)	2 (5.0)	8 (17.0)		
ypN	0	51 (69.9)	48 (72.7)	51 (69.9)	55 (66.3)	NaN	
	[1-3]	18 (24.7)	15 (22.7)	13 (17.8)	24 (28.9)		
	[4-9]	4 (5.5)	3 (4.5)	9 (12.3)	4 (4.8)		
	10 and more	0 (0.0)	0 (0.0)	0 (0.0)	0 (0.0)		
Stromal TIL levels (%) (post-NAC)		10.00 [5.00, 15.00]	10.00 [5.00, 15.00]	7.50 [4.50, 11.25]	10.00 [5.00, 15.00]	0.619	nonnorm
Stromal TIL levels (%) (post-NAC)	[0,10]	32 (68.1)	29 (69.0)	30 (75.0)	33 (70.2)	0.904	
	(10,100]	15 (31.9)	13 (31.0)	10 (25.0)	14 (29.8)		
IT TIL levels (%) (post-NAC)		5.00 [2.00, 10.00]	7.50 [5.00, 10.00]	5.00 [2.00, 10.00]	5.00 [2.00, 15.00]	0.449	nonnorm
IT TIL levels (%) (post-NAC)	[0,10]	22 (78.6)	18 (90.0)	23 (85.2)	21 (70.0)	0.306	
	(10,100]	6 (21.4)	2 (10.0)	4 (14.8)	9 (30.0)		

Supplementary Table S1. *Patients 'characteristics according to season in each tumor subtypeon .*

	Class	n total	n in model	nb pcr	%pcr	OR	95%CI	pval
Age at BC diagnosis				292		1	[0.99 - 1.02]	0.589
Menopausal status	Premenopausal	747	744	173	23.3 %	1		
	Postmenopausal	442	440	116	26.4 %	1.18	[0.9 - 1.55]	0.229
BMI	18.5-24.9	681	680	171	25.1 %	1		
	<18.5	48	45	7	15.6 %	0.55	[0.22 - 1.18]	0.153
	25-29.9	304	304	72	23.7 %	0.92	[0.67 - 1.26]	0.623
	>=30	160	158	39	24.7 %	0.98	[0.65 - 1.45]	0.904
Smoking status	Never	623	621	172	27.7 %	1		
	Current	179	179	36	20.1 %	0.66	[0.43 - 0.98]	**0.042**
	Former	154	154	45	29.2 %	1.08	[0.73 - 1.58]	0.706
Year BC diagnosis	2002	89	88	10	11.4 %	1		
	2003	62	62	5	8.1 %	0.68	[0.2 - 2.04]	0.509
	2004	101	99	20	20.2 %	1.97	[0.89 - 4.65]	0.104
	2005	127	125	30	24%	2.46	[1.17 - 5.59]	**0.023**
	2006	143	143	27	18.9 %	1.82	[0.86 - 4.13]	0.134
	2007	148	148	35	23.6 %	2.42	[1.17 - 5.41]	**0.023**
	2008	151	151	40	26.5 %	2.81	[1.37 - 6.25]	**0.007**
	2009	170	170	48	28.2 %	3.07	[1.52 - 6.75]	**0.003**
	2010	137	136	53	39%	4.98	[2.46 - 11.01]	**<0.001**
	2011	60	60	19	31.7 %	3.61	[1.57 - 8.78]	**0.003**
	2012	11	11	5	45.5 %	6.5	[1.62 - 25.8]	**0.007**
Season of BC diagnosis	Winter	304	303	71	23.4 %	1		
	Spring	288	287	75	26.1 %	1.16	[0.8 - 1.68]	0.448
	Summer	281	279	66	23.7 %	1.01	[0.69 - 1.49]	0.949
	Fall	326	324	80	24.7 %	1.07	[0.74 - 1.55]	0.713
Hereditary predisposition	No	221	219	64	29.2 %	1		
	Yes	46	46	21	45.7 %	2.03	[1.06 - 3.89]	**0.032**
Clinical T stage (TNM)	T0-T1	70	70	32	45.7 %	1		
	T2	798	795	192	24.2 %	0.38	[0.23 - 0.62]	**<0.001**
	T3-T4	330	327	68	20.8 %	0.31	[0.18 - 0.54]	**<0.001**
Clinical N stage (TNM)	N0	525	524	126	24%	1		
	N1-N2-N3	673	668	166	24.9 %	1.04	[0.8 - 1.36]	0.749
SBR grade	Grade I	47	47	1	2.1 %	1		
	Grade II	432	428	58	13.6 %	7.21	[1.53 - 128.94]	0.053
	Grade III	678	677	225	33.2 %	22.9	[4.96 - 406.92]	**0.002**
KI67	[0-10)	65	65	3	4.6 %	1		
	[10-20)	110	110	9	8.2 %	1.84	[0.53 - 8.54]	0.373
	>=20	407	403	105	26.1 %	7.28	[2.63 - 30.25]	**0.001**
Mitotic index	[0-7) mitose/2 mm2	341	338	45	13.3 %	1		
	[7-13) mitose/2 mm2	295	294	67	22.8 %	1.92	[1.27 - 2.93]	**0.002**
	>=13 mitose ou plus/2 mm2.	445	445	152	34.2 %	3.38	[2.35 - 4.93]	**<0.001**
BC subtype	Luminal	528	526	34	6.5 %	1		
	TNBC	376	374	143	38.2 %	8.96	[6.04 - 13.62]	**<0.001**
	HER2+	295	293	115	39.2 %	9.35	[6.21 - 14.4]	**<0.001**
DCIS component	No	604	602	180	29.9 %	1		
	Yes	389	388	76	19.6 %	0.57	[0.42 - 0.77]	**<0.001**
Stromal TIL levels (%)	[0 -30[	475	473	103	21.8 %	1		
	>=30	242	242	106	43.8 %	2.8	[2 - 3.92]	**<0.001**
IT TIL levels (%)	[0,10]	511	509	124	24.4 %	1		
	(10,100]	206	206	85	41.3 %	2.18	[1.55 - 3.07]	**<0.001**
LVI	No	267	266	48	18%	1		
	Yes	171	170	33	19.4 %	1.09	[0.66 - 1.78]	0.721
Histological type	NST	1062	1058	271	25.6 %	1		
	Others	74	73	5	6.8 %	0.21	[0.07 - 0.48]	**0.001**

Supplementary Table S2. *Association of season with pCR after univariate analysis in the whole population.*

Supplementary Table S3. *Literature Review*

Authors	Country	Journal	Type of source	Research design	Number of Patients (n)	Study population	Summary points
Cohen (1983)	Israel	Cancer Research	Research	Registry	2.948	Breast cancer with histopathological confirmation	There was a seasonal variation in the incidence of nonlocalized breast cancer diagnosis, with a peak during spring.
Holdaway (1990)	New Zeland	Cancer Research	Research	Prospective Cohort	2.706	Breast cancer	According to the month of initial tumor detection, tissue sampling, or surgery, there was a significant annual variation in PR receptor concentration. PR receptor concentrations were higher in patients diagnosed or operated on during April. There is also a significant cyclic variation in the month of detection of breast cancer with peak detection occurring in December (summer).
Mason (1990)	New Zealand	British Journal of Cancer	Research	Prospective Cohort	2.245	Incidente breast cancer	Breast cancer diagnoses in spring and summer were associated with better survival. These patterns have been different according to the menopausal status and hormonal receptors. Postmenopausal women with ER positive tumours had 13% improvement in survival at 5 years if the initial tumour was detected in spring/summer ($p < 0.0003$, OR 2.04 [CI 1.38-3.01]). Premenopausal women with HR positive tumours there was no significant relationship between season of detection and survival. However, premenopausal patients had improved survival (26% at 5 years) if they had HR tumours which were found initially in the spring/summer.
Joensuu (1991)	Finland	British Journal of Cancer	Research	Retrospective Cohort	401	Invasive, non bilateral breast cancer patients	Cancers diagnosed during January, February, or July-August to September-October had a poorer prognosis, further tumor necrosis, advanced mitotic count, and larger tumor size than if the diagnosis was made during the rest of the year ($p = 0.03$ and 0.009, respectively).
Ross (1997)	USA	Breast Cancer Research and Treatment	Short Communication	Case-control study	2.895	Breast cancer self-detected that required surgery	There was a statistically significant seasonal variation in the detection of BC, with peaks occurring in spring and late fall. Women under 40, had a spring peak detection ($p = 0.089$) and women ≥40 in late fall ($p = 0.063$).The seasonal variation in detection was statistically significant only for the estrogen receptor-negative tumors ($p = 0.003$).
Gao (2001)	Singapure	British Journal of Cancer	Short Communication	Registry	3.219	Breast cancer, histologically confirmed	There is no clear cut seasonal pattern, although fewer cases are diagnosed in January and February (close to the Western and Chinese New Year festivals). They appeared more numerous approximately six months later over the June to August period. Also, there were more ER+/PR+ BC in October, and tumors were bigger (≥1 cm) if diagnoses were made in August.
Paradiso (2001)	Italy	Breast Cancer Research and Treatment	Research	Retrospective Cohort	903	T1-3, N0-1M0 Invasive breast cancer, treated with radical mastectomy or lumpectomy and lymphadenectomy	There were no differences in nodal status or tumor size, ER, and PgR across seasons in the general population. Grade III tumors were significantly more frequent in spring. These patterns have been different according to the menopausal status of the patient. In premenopausal BC patients, ER had a peak in January and April; PgR in July ($p < 0.05$). Also, tumor size had peaks in May and December ($p < 0.01$). In menopausal patients, ER had a rise in November, no significant reproducible rhythm was reported for PgR, and tumor size had a peak in September.
Lambe (2003)	Sweden	British Journal of Cancer	Short Communication	Registry	60.143	Breast cancer	There was a seasonal variation in breast cancer detection, decreasing during the summer (-16.7%).
Lim (2006)	United Kingdom	International Journal of Cancer	Research	Registry	182.895	Breast cancer	Breast cancer diagnoses in summer and autumn were associated with better survival compared to breast cancer diagnoses in winter (HR 0.86 [95% CI 0.83–0.89]).
Porojnicu (2007)	Norway	Breast Cancer Research and Treatment	Research	Registry	49.821	Breast cancer	Breast cancer diagnoses in summer had better prognosis (RR of death 15–25% lower for summer diagnosis versus winter diagnosis). There was no significant seasonal variation of the number of new cases.
Roychoudhur (2008)	United Kingdom	International Journal of Cancer	Research	Registry	187.263	Breast cancer	Diagnosis within the summertime season becomes related to significantly reduced mortality in the first month of diagnosis compared with winter(HR 0.81 [95% CI, 0.75–0.86]). However, at long-time follow-up (>5 years), there has been a steady shift within the seasonality pattern, with an autumn diagnosis related to reduced mortality (HR 0.93 [95% CI, 0.90–0.96]).

Authors	Country	Journal	Type of source	Research design	Number of Patients (n)	Study population	Summary points
Holmberg (2009)	Sweden	Cancer Causes Control	Research	Registry	89.630	Breast cancer	There was a higher HR of death in women diagnosed with BC in the summer (August vs January, HR 1.14 [95% CI, 1.09–1.19]). This difference coincided with a lower mean number of cases diagnosed per day, and a higher proportion of advanced cases diagnosed in the summer.
Stajner (2009)	Atlanta, Connecticut, Detroit, Hawaii, Iowa, New Mexico, San FranciscoOakland, Seattle-Puget Sound, and Utah	International Journal of Cancer	Letter to editor	Registry	184.434	Breast cancer	After 12 years of follow up, BC diagnosed in winter between 1980–1986 had the highest cumulative probability of death [HR 0.32 [95% CI, 0.315–0.33] and the lowest if cases diagnosed in autumn (HR 0.29[95% CI,0.29–0.30]) and spring (HR 0.30[95% CI, 0.29–0.31]). However, for each of the other 2 periods (1973–1979 and 1987–1993) prognosis from BC was independent of the seasonality of the diagnosis.
Oh (2010)	International study (88 cancer registries)	Breast Cancer Research and Treatment	Research	Registry	2.921.714	Breast cancer	Breast cancer is diagnosed more often in spring and fall, both in the Northern and Southern Hemispheres. This seasonality is increasingly more prominent as population distance from the equator increases and this latitude dependence is most pronounced among women living in rural areas.
Teilum (2011)	Denmark	British Medical Journal Open	Research	Registry	79.658	Early invasive breast cancer that required surgery	There were no evidence of a seasonal variation in the survival after surgery for early BC
Kwon (2013)	Korean	Korean Journal of Clinical Oncology	Research	Registry	11.698	Breast cancer	There were no differences in patient characteristics and ER, PR, tumor size, and lymph node involvement among the breast cancers diagnosed in spring/ summer vs. autumn/winter, except for age (p< 0.01) and HER2 status (p= 0.02). Also, there were no differences in survival and relapse rates.
Mutlu (2013)	Turkey	Asian Pacific Journal of Cancer Prevention	Research	Retrospective Cohort	517	Breast cancer	It was not found any stastically significant differences among groups regarding age, menopause status, stage of breast cancer, ER, PgR, Her2+, grade, lymphovascular invasion. Patients whose were diagnosed in winter had best mean DFS while summer group had worst DFS. When we evaluated mean OS, we did not find any differences (p=0.637).
Ho (2014)	USA	Medical Science Monitor	Research	Registry	19.204	Breast cancer	BC diagnoses in summer had better prognosis than BC diagnoses in winter at all latitudes (HR 0.94 [95% CI, 0.94 to 0.94], p = 0.002).
Kuzmickiene (2018)	Lithuania	Journal of Cancer Research and Therapeutics	Research	Retrospective Cohort	991	Non-metastatic breast cancer patients	BC diagnosis in summer and autumn had a 40% reduced risk for 0–3-year mortality when compared to those diagnosed in spring. The impact season of diagnosis on mortality was most pronounced in young patients. BC diagnosis in autumn is an independent predictor factor for better overall prognosis in women under the age of 50 years (HR = 0.61[95% CI,0.39–0.96, p = 0.003).
Tas (2021)	Turkey	Indian Journal of Surgery	Letter to editor	Retrospective Cohort	5.477	Breast cancer	The number of newly diagnosed breast cancer patients is significantly lower in summer than in any other season (summer (22,0%) vs spring (27.0%), autumn (25.6%) and winter (25.3%)).
Wu (2021)	USA	Journal of Clinical Medicine	Research	Prospective Cohort	2.919	Primarily early stage breast cancer	Blood collected in the winter season was associated with lower C-reactive (CRP) protein when compared to summer. In addition, moderate intakes of red meat were associated with reducing CRP in winter but not in other seasons. Increased intakes of fruit and vegetables were associated with reduced inflammation in most seasons except winter.
Yoon (2021)	Korea	Journal of Gastroenterology and Hepatology	Research	Retrospective Cohort	1.313	Breast cancer	There was a winter peak for breast cancer detection (72.7–76.3%) higher than for other cancers: thyroid cancer (44.1–59.4%), lung cancer (36.8–41.7%), kidney cancer (34.141.2%), and prostate cancer (27.7–34.7%).

Supplementary Table S3. *Literature Review (continuation)*
